# The Leeds Evaluation of Efficacy of Detoxification Study (LEEDS) prisons project pilot study: protocol for a randomised controlled trial comparing dihydrocodeine and buprenorphine for opiate detoxification

**DOI:** 10.1186/1745-6215-8-1

**Published:** 2007-01-08

**Authors:** Laura Sheard, Clive E Adams, Nat MJ Wright, Hany El-Sayeh, Richard Dalton, Charlotte NE Tompkins

**Affiliations:** 1 Leeds West Primary Care Trust based at Centre for Research in Primary Care, 71-75 Clarendon Road, Leeds, LS2 9PL, UK; 2 Department of Psychiatry, 15 Hyde Terrace, University of Leeds, Leeds, LS2 9LT, UK; 3 Craven, Harrogate and Rural District Primary Care Trust, The Briary Wing, Harrogate District Hospital, Lancaster Park Road, Harrogate HG2 7SX, UK; 4 Formerly HMP Leeds, 2 Gloucester Terrace, Armley, Leeds, LS12 2TJ, UK

## Abstract

**Background:**

In the United Kingdom (UK), there is an extensive market for the class 'A' drug heroin. Many heroin users spend time in prison. People addicted to heroin often require prescribed medication when attempting to cease their drug use. The most commonly used detoxification agents in UK prisons are buprenorphine, dihydrocodeine and methadone. However, national guidelines do not state a detoxification drug of choice. Indeed, there is a paucity of research evaluating the most effective treatment for opiate detoxification in prisons. This study seeks to address the paucity by evaluating routinely used interventions amongst drug using prisoners within UK prisons.

**Methods/Design:**

The Leeds Evaluation of Efficacy of Detoxification Study (LEEDS) Prisons Pilot Study will use randomised controlled trial methodology to compare the open use of buprenorphine and dihydrocodeine for opiate detoxification, given in the context of routine care, within HMP Leeds. Prisoners who are eligible and give informed consent will be entered into the trial. The primary outcome measure will be abstinence status at five days post detoxification, as determined by a urine test. Secondary outcomes during the detoxification and then at one, three and six months post detoxification will be recorded.

## Background

In the United Kingdom (U.K), there is an extensive market for the sale of heroin, an illicit class 'A' drug. Precise figures of how many people use and are dependent on heroin are difficult to establish as there has never been a national prevalence survey [1]. The link between crime and illicit drug use, specifically heroin use, is well recognised. In 2002, 63% of injecting drug users (IDUs) in contact with specialist drugs services in England and Wales reported having ever been in prison or a young offenders' institution [2]. Those addicted to illicit opiates such as heroin require medical help in reducing and stopping their use due to the drug's highly addictive properties.

Neither the current evidence base [1] nor the national guidelines on the clinical management of drug misuse [3] stipulate a 'drug of choice' for rapid opiate detoxification. In the absence of an established evidence base, a wide variety of agents have therefore been used including methadone, dihydrocodeine, buprenorphine, lofexidine and clonidine. Historically within prisons, the most commonly used drug for opiate detoxification has been dihydrocodeine. Anecdotally this was because of a reluctance to prescribe methadone following a small number of methadone related deaths in the prison setting. Dihydrocodeine is attractive to clinicians as it has a shorter half-life than methadone, and seems equally acceptable to users. However, there has been a move away from prescribing dihydrocodeine due to its potential for diversion by prisoners into the prison shadow economy. Increasingly, buprenorphine is now being prescribed and recent policy supports methadone prescribing [4]. Buprenorphine – in the form of sub-lingual tablets – is relatively new for the purpose of opiate detoxification in the UK, especially within the prison estate. It has the potential advantage of having a good safety profile, better retention in treatment and lower withdrawal severity when compared to methadone, lofexidine or clonidine [5-8]. Her Majesty's Prison (HMP) Leeds saw a 42% increase in buprenorphine prescribing between April 2002 and January 2004. Buprenorphine is increasingly being prescribed in the community [9].

There appears to be a paucity of clinical trials conducted in the UK prison setting which have evaluated medication for opiate detoxification. Whilst one study evaluated the withdrawal severity of methadone and lofexidine [10], the rates of completion were not sufficient to detect a statistically significant difference between the two medications. One study has compared sublingual buprenorphine with dihydrocodeine for postoperative pain, outside of the prison setting [11]. In 2004, the Leeds Evaluation of Efficacy of Detoxification Study (LEEDS) team conducted the first randomised controlled trial (RCT) to compare buprenorphine and dihydrocodeine for opiate detoxification in the community [12]. The results have recently been submitted for publication. The LEEDS trial has since expanded to evaluate the open use of buprenorphine versus dihydrocodeine in the prison primary healthcare setting.

## Methods

### Design

LEEDS is a pragmatic open label randomised controlled trial.

Randomisation will be random block size, which will be administered centrally in the Academic Department of Psychiatry and Behavioural Sciences, University of Leeds. Opaque consecutively numbered envelopes will be prepared. If a prisoner is both eligible and agreeable the next envelope will be opened and the intervention allocated.

Confounding variables i.e. doctor participant relationships, drugs worker participant relationships will all be controlled for by randomisation. Some confounding variables cannot be randomised. These include the inherent difference in the two detoxification programmes, i.e. the daily supervised consumption of buprenorphine.

### Setting and recruitment

The study will take place in prison primary healthcare at HMP Leeds, West Yorkshire, England. HMP Leeds is a category B local prison, which accepts all adult male prisoners aged 21 and over from West Yorkshire. Participants will be recruited from the medical reception area on first arrival into the prison, where prisoners are routinely offered at detoxification regime.

LEEDS is intended to complement rather than compete with research taking place in a community primary or secondary care setting. Much of the current evidence base for pharmacological treatment of drug use comes from studies that have taken place in an inpatient or residential setting. There is an urgent need to verify whether such research is applicable to the prison primary healthcare environment. The ethos of the study will be a collaborative project between prison primary healthcare, primary care and the secondary care academic departments and service providers.

### Sample size

Currently no randomised controlled trials relevant to these comparisons have been undertaken in the prison setting. However, from the results of our community study comparing these agents we estimate that with a sample size of 120 we will have a finding of clinical and statistical significance. Power calculations were undertaken using Sample Power 1.20 developed by SPSS Inc., comparing two groups (60 individuals in each) and for α = 0.05 (two-sided). The power calculations are shown below in Table 1.

**Table 1:** Power calculations for sample of 120Difference between groups

	**Power**	**Difference between groups**	**Power**
70% × 60%	21%	40% × 30%	21%
70% × 50%	61%	40% × 20%	67%
70% × 45%	80%	40% × 15%	88%
70% × 40%	92%		
60% × 50%	20%	30% × 20%	24%
60% × 40%	59%	30% × 15%	50%
60% × 35%	79%	30% × 10%	79%
60% × 30%	92%		
50% × 40%	20%	20% × 15%	11%
50% × 30%	61%	20% × 10%	33%
50% × 25%	82%	20% × 5%	70%

### Eligibility

#### Inclusion criteria

1. Male or female

2. 18 – 65 years old

3. Using illicit opiates as confirmed by a urine test taken at first assessment.

4. Expressing a wish to detoxify through the standard monitored process and remain abstinent from opiates.

5. Willing to give informed consent after receiving the participant information booklet

6. Remaining in custody for longer than 28 days

#### Exclusion criteria

1. Contraindications to methadone or buprenorphine

2. Co-existing acute medical conditions requiring emergency admission for hospital care so precluding detoxification in the prison setting

3. Currently undergoing detoxification from other addictive drugs whereby concurrent detoxification from opiates would not be clinically indicated

4. Previously been randomised into this trial

### Interventions

The treatment option will be concealed from both participant and clinician at time of assessment. Both parties will remain blind to the selected treatment option until the envelope is opened.

1. Dihydrocodeine, given openly, in the context of the standard prison doctor and drugs worker support.

2. Buprenorphine, given openly, in the context of the standard prison doctor and drugs worker support.

The reducing regimen of both dihydrocodeine and buprenorphine (over less than 16 days) will be at the discretion of the prescribing doctor. However, the dose should not exceed this standard regime (Table 2 and Table 3).

**Table 2:** Buprenorphine detoxification

**Day**	**Dose (mg)**
1	6
2	8
3	8
4	6
5	6
6	4
7	3.6
8	3.2
9	2.8
10	2.4
11	2.0
12	1.6
13	1.2
14	0.8
15	0.4

**Table 3:** Dihydrocodeine detoxification

Day	Morning	Evening
1	XXXX	2 × 120 mg
2	2 × 120 mg	2 × 120 mg
3	1 × 120 mg 1 × 90 mg	1 × 120 mg 1 × 90 mg
4	1 × 120 mg 1 × 90 mg	1 × 120 mg 1 × 90 mg
5	2 × 90 mg	2 × 90 mg
6	2 × 90 mg	2 × 90 mg
7	1 × 90 mg 1 × 60 mg	1 × 90 mg 1 × 60 mg
8	1 × 90 mg 1 × 60 mg	1 × 90 mg 1 × 60 mg
9	2 × 60 mg	2 × 60 mg
10	2 × 60 mg	2 × 60 mg
11	1 × 60 mg	2 × 60 mg
12	1 × 60 mg	2 × 60 mg
13	1 × 60 mg	1 × 60 mg
14	1 × 60 mg	1 × 60 mg
15	XXXX	1 × 60 mg

### Outcomes

#### Primary outcome

Abstinence from street opiates at five days post detox as indicated by urine test. Should the person not finish the course of detoxification or refuse to give urine then this will be considered as a positive urine test for opiates.

#### Secondary outcomes

##### During the period of detoxification

###### Adverse events

Clinicians will record any details of adverse events in the usual way i.e. an entry will be made in the participants' records. Those adverse events clearly resulting in clinically significant distress to study participants or of major concern to clinicians will be recorded. The LEEDS trial co-ordinator will extract data from clinical records, for the period of detoxification, and transcribe these to a database.

###### Leaving the study early

Perceived reasons for withdrawal will be recorded.

###### Inappropriate use of prescribed medication

Examples of this include intentional overdose and storing, trading, swapping or selling of prescribed medication.

###### Service related outcomes

Admission to hospital, Accident and Emergency and in patient stays in prison healthcare will be recorded.

##### At one-month, three-month and six-month post detoxification

###### Abstinence status

Evidence of this taken from clinical notes if the participant is still in HMP Leeds or has been transferred to another prison. If the participant has been released into the community then they will be contacted via address or telephone number given at point of randomisation, or by accessing local community drugs service records.

###### Service utilisation

Contacts with hospitals and Accident and Emergency departments will be noted. For those participants still in the prison estate, use of inpatient prison healthcare will be recorded.

The project team recognise the difficulty in tracking people with drug problems who have subsequently been released from prison across this period.

#### Data collection

The outside of the allocation envelope will be completed by the prison doctor before opening. This will record Unit number, date of birth and estimate of severity of addiction and expected prognosis.

The LEEDS trial co-ordinator will collect details of drug of allocation, background history, and use of opiates from the participant's health records. The primary outcome and data at one, three months and six months after randomisation will again be collected by the LEEDS trial co-ordinator. There will be minimal additional contact with the participants. LEEDS is designed to avoid the complication of the care provided to this group.

All data will be double entered into an EPI-INFO database customised for the LEEDS project and the simple analysis involved with this project will also be undertaken in that package.

#### Analysis

After data entry, all analysis will be undertaken in Epi-info. The analysis of the primary outcomes will be of a simple 2 × 2 table (see Table 4, below). Dummy tables have been constructed for all secondary outcomes. These tables are designed to be rigid templates for the final write up of the project, and to protect the researchers from bias, once the data are disclosed. An example is given below, but dummy tables for all data to be collected have been constructed, *a priori*, for every variable to be collected.

**Table 4:** Dummy table for abstinence from street heroin at final prescription as indicated by urine test

	Abstinence successful	Abstinence not successful	Totals
Buprenorphine	A	C	A+C
Dihydrocodeine	B	D	B+D
Totals	A+B	C+D	A+B+C+D

RR XX 95% CI XX-XX, NNT XX 95% CI XX-XX

## Discussion

The information which the LEEDS project team aim to gain from the trial is twofold. Firstly, once data collection is complete, we hope to show either a statistically significant difference between the efficacy of the two detoxification agents or alternatively, that no statistically significant difference exists. We expect that the results will influence prison healthcare policy if the trial demonstrates one detoxification agent is significantly more efficacious than another. This may begin locally within HMP Leeds and potentially influence prison prescribing policy nationally.

Information about the methodological practicalities of conducting a randomised controlled trial in the prison setting will be enormously valuable. The research team have previously described the practicalities of conducting a detoxification RCT in primary care [13]. Yet, to our knowledge, only one British prison RCT comparing detoxification agents exists [10]. Therefore, conducting this current trial will provide a wealth of previously unknown knowledge about the feasibility, practicality and day to day groundwork and management involved in running a trial in the prison environment. This information may be important for future research teams, and will also demonstrate that trials can be done within prisons.

Given the factors above, the importance of this study cannot be under estimated. Guidelines for substance misuse treatment in prison are not grounded on an established evidence base as there is an extreme paucity of trials conducted in this setting. Consequently, the project team envisage that this trial will provide invaluable data about the efficacy of two routinely used detoxification agents which have rarely been studied within the prison environment.

### Approvals process

Research Governance approval was granted by Bradford South and West Primary Care Trust (PCT) on 20^th ^April 2004. Research ethics approval was granted from a Multi Centre Research Ethics Committee (MREC) Wales on 14^th ^May 2004.

### Consent

Consent for the participant to enter the trial will be gained at first presentation to the prison doctor once eligibility criteria have been confirmed. Consent will be obtained in the form of written consent after the participant has made an informed decision. Information will be provided to the participant using an information leaflet documenting the aims, objectives and necessity of the study, which has been approved by the research ethics committee (MREC Wales).

### Funding

Funding for this project has been acquired from Leeds Primary Care Trusts Research Consortium Priority and Needs Funding.

## Competing interests

The author(s) declare that they have no competing interests.

## Authors' contributions

NW and CA designed the study, offer project supervision and drafted the proposal. NW is principal investigator. CA centrally manages the randomisation process and will conduct statistical analysis. LS co-ordinates and manages the project, assists during randomisation clinics, collects data and drafted the manuscript. HE and NW randomise participants into the trial. RD collects data and drafted the manuscript. CT drafted the manuscript.
